# Analgesic Effect of Tranilast in an Animal Model of Neuropathic Pain and Its Role in the Regulation of Tetrahydrobiopterin Synthesis

**DOI:** 10.3390/ijms23115878

**Published:** 2022-05-24

**Authors:** Swarnalakshmi Raman, Arief Waskitho, Resmi Raju, Takuma Iwasa, Daisuke Ikutame, Kazuo Okura, Masamitsu Oshima, Yoshizo Matsuka

**Affiliations:** 1 Department of Stomatognathic Function and Occlusal Reconstruction, Graduate School of Biomedical Sciences, Tokushima University, Tokushima 770-8504, Japan; swar.raman@gmail.com (S.R.); arief.waskitho.85@gmail.com (A.W.); iwasatakuma0904@outlook.jp (T.I.); c000030613@tokushima-u.ac.jp (D.I.); okura.kazuo@tokushima-u.ac.jp (K.O.); m-oshima@tokushima-u.ac.jp (M.O.); 2 Eunice Kennedy Shriver National Institute of Child Health and Human Development, Bethesda, MD 20892, USA; resmi.raju@nih.gov

**Keywords:** orofacial pain, neuropathic pain, infraorbital nerve constriction, nerve injury, tetrahydrobiopterin, tranilast, trigeminal ganglion

## Abstract

Trigeminal neuralgia is unilateral, lancinating, episodic pain that can be provoked by routine activities. Anticonvulsants, such as carbamazepine, are the drugs of choice; however, these possess side-effects. Microvascular decompression is the most effective surgical technique with a higher success rate, although occasionally causes adverse effects. The potential treatment for this type of pain remains unmet. Increased tetrahydrobiopterin (BH4) levels have been reported in association with axonal injury. This study aimed to evaluate the effect of tranilast on relieving neuropathic pain in animal models and analyze the changes in BH4 synthesis. Neuropathic pain was induced via infraorbital nerve constriction. Tranilast, carbamazepine, or saline was injected intraperitoneally to assess the rat’s post-intervention pain response. In the von Frey’s test, the tranilast and carbamazepine groups showed significant changes in the head withdrawal threshold in the ipsilateral whisker pad area. The motor coordination test showed no changes in the tranilast group, whereas the carbamazepine group showed decreased performance, indicating impaired motor coordination. Trigeminal ganglion tissues were used for the PCR array analysis of genes that regulate the BH4 pathway. Downregulation of the sepiapterin reductase (*Spr*) and aldoketo reductase (*Akr*) genes after tranilast injection was observed compared to the pain model. These findings suggest that tranilast effectively treats neuropathic pain.

## 1. Introduction

Orofacial pain is a collective term used to represent pain affecting the face and/or oral cavity [1]. Peripheral nerve injury leads to the production of high-frequency injury signals in injured nerve fibers. Injury to the trigeminal system results in the transmission of injury signals to the trigeminal spinal subnucleus caudalis and upper cervical spinal cord (C1–C2) via the trigeminal ganglion (TG), leading to orofacial pain [2]. However, the mechanism underlying trigeminal neuropathic pain remains unclear [3]. Medication is the first-line treatment for this condition. The commonly prescribed drugs are anticonvulsants, such as carbamazepine (gold standard), which has been reported to provide partial pain relief in about 80 to 90% of the patients. The most frequent side-effects of carbamazepine include dizziness, drowsiness and nausea. Other drugs used include oxcarbazepine, pregabalin, gabapentin, phenytoin, lamotrigine and baclofen [4]. In addition to these drugs, non-steroidal anti-inflammatory medications (NSAIDs) have also been prescribed in routine practice. However, they are ineffective in treating neuropathic pain due to their limited efficacy [5]. Surgical options include microvascular decompression (MVD), stereotactic radiosurgery and peripheral surgical techniques, such as neurectomy and cryotherapy. MVD is often the preferred technique, and it has a prolonged duration of pain relief. However, surgical management may cause dysesthesia, the most commonly reported adverse effect [6].

Despite the multitude of therapeutic options, trigeminal neuralgia management remains challenging and unsatisfactory due to inter-patient variations. Complete pain abolition is rare, and the best approach is to minimize pain and improve quality of life. The lack of clear mechanisms is the foremost reason for the inability to cure neuropathic pain [7]. Several novel potential therapeutic targets have been investigated, including transient receptor potential ankyrin 1 (TRPA1) antagonist, peroxisome proliferator-activated receptor gamma (PPARγ) antagonists, and extracellular signal-regulated kinase (ERK) and jun N-terminal kinase (JNK) mitogen-activated protein kinase (MAPKs) inhibitors [8]. However, these new targets are still in the preclinical stages and require several phases of clinical trials to be approved for use in humans. A potential option is the use of previously approved drugs. One such drug is tranilast, a therapeutic drug that has been reported for repurposing in neuropathic pain treatment, in an in vitro study [9].

Tranilast (N-3′,4′- dimethoxycinnamoyl-anthranilic acid) is a tryptophan metabolite. It has been in use since the 1980s and was primarily invented for treating allergic conditions, such as asthma, allergic conjunctivitis, and keloids [10]. The ability of tranilast to inhibit the release of chemical mediators from the mast cells was thought to be the mechanism of action in therapeutic application as an anti-allergic drug [11]. Furthermore, the effectiveness of tranilast has been established in autoimmune diseases, cardiovascular diseases and even in several in vivo studies of various cancers, such as prostate cancer, breast cancer, pancreatic cancer and oral squamous cell carcinoma [12].

The association between tranilast and pain relief has been reported in a previous study wherein pelvic pain was reduced; however, the mechanism of action was not established [13]. The anti-inflammatory and analgesic properties of tranilast in the treatment of rheumatoid arthritis was reported previously [14]. These studies suggest that tranilast may possess an analgesic property and may be effective in treating neuropathic pain. An earlier in vitro study not only suggested the effect of tranilast in treating neuropathic pain but also demonstrated the binding site of the drug in the pockets of sepiapterin in a molecular docking model [9]. Sepiapterin is an organic compound that is metabolized into tetrahydrobiopterin (BH4). BH4 is formed via three enzymatic pathways: de novo, salvage and recycling. It is an essential cofactor in the biosynthesis of monoamine neurotransmitters. BH4 plays a critical role in cardiovascular function, neurotransmission, mood and inflammation [15,16]. Increased production of BH4 has been reported to be associated with axonal injury, which is an ideal target for the treatment of neuropathic pain [17]. To date, no drugs have been reported to alleviate neuropathic pain through BH4 regulation. In this study, we aimed to evaluate the analgesic effect of tranilast in relieving neuropathic pain and analyze the changes in the gene regulation involved in BH4 synthesis using TG.

## 2. Results

### 2.1. Infraorbital Nerve-Constriction-Induced Pain Behavior

**Figure 1:**
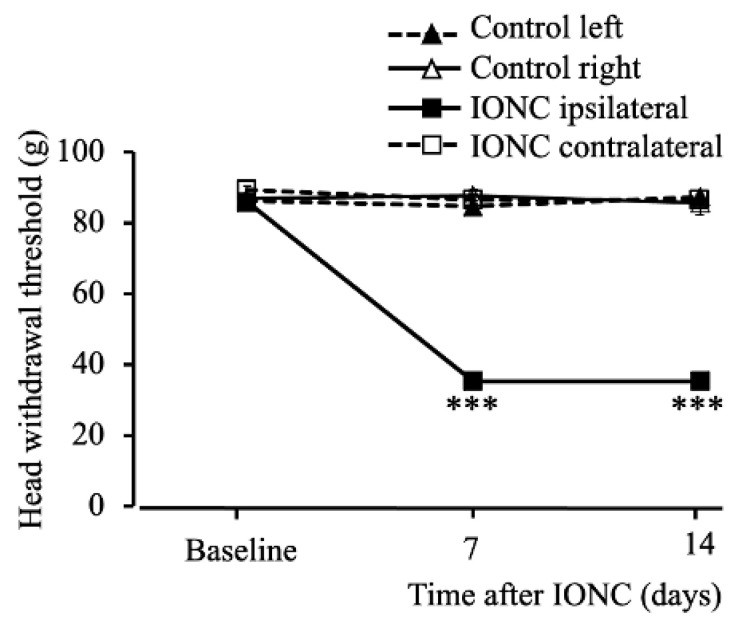
Head withdrawal threshold to mechanical stimuli. Bilateral head withdrawal threshold to mechanical stimuli in whisker pad area on day 7 and 14 after surgery (infraorbital nerve constriction (IONC)). In the IONC group, head withdrawal thresholds were significantly decreased in the ipsilateral side after day 7 and 14 of surgery compared on to the contralateral side. The head withdrawal thresholds are represented as mean ± standard error of the mean (SEM). *n* = 6 per group. *** *p* < 0.001, repeated measures analysis of variance followed by Tukey’s HSD test.

The head withdrawal threshold as a response to mechanical stimuli was recorded at baseline (ipsilateral: 85.93 ± 0.71 g and contralateral: 89.22 ± 1.24 g (mean ± standard error of the mean (SEM))). There was a significant decrease in the pain threshold in the infraorbital nerve constriction (IONC) group on days 7 (35.29 ± 1.41 g) (F _(3,20)_ = 317.44), *p* < 0.001) and 14 (35.25 ± 0.77 g) (F _(3,20)_ = 157.67), *p* < 0.001) in the ipsilateral whisker pad area (Figure 1).

### 2.2. Dose Dependent Effect of Tranilast to Mechanical Stimuli Post IONC

**Figure 2:**
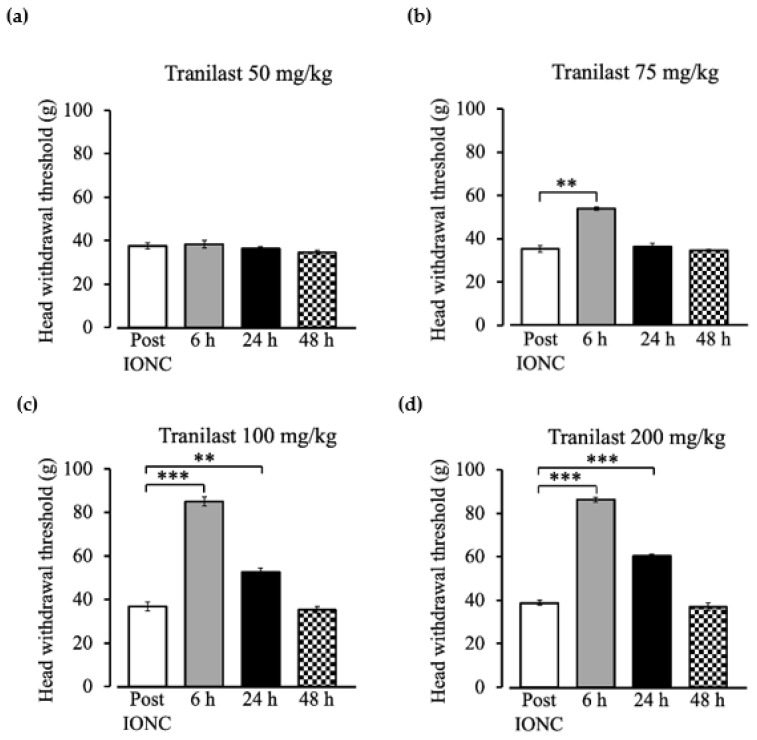
Head withdrawal threshold to mechanical stimuli after intraperitoneal injection of tranilast. (**a**) Tranilast 50 mg/kg showed no changes in head withdrawal threshold to mechanical stimuli. (**b**) Tranilast 75 mg/kg dosage increased the threshold to mechanical stimuli at 6 h post injection. (**c**) Tranilast 100 mg/kg and (**d**) tranilast 200 mg/kg dosages increased the threshold to mechanical stimuli at 6 and 24 h in comparison to the pain model threshold. The head withdrawal thresholds are represented as mean ± SEM. *n* = 6 per group. ** *p* < 0.01, *** *p* < 0.001, repeated measures analysis of variance followed by Tukey’s HSD test.

Intraperitoneal injection of tranilast 50 mg/kg did not result in any changes in the head withdrawal threshold (Figure 2a). A significant change in head withdrawal threshold was observed at 6 h (53.89 ± 0.73 g) following the tranilast injection of 75 mg/kg (Figure 2b, F _(3,15)_ = 98.5, *p* = 0.001), and no effect was observed at 24 h (36.32 ± 1.54 g) (Figure 2b, F _(3,15)_ = 98.5, *p* = 1) when compared to the pain model (35.37 ± 1.49 g). A dose of 100 mg/kg (Figure 2c, F _(3,15)_ = 331.43, *p* < 0.001) and 200 mg/kg (Figure 2d, F _(3,15)_ = 65.59, *p* < 0.001) showed an increased head withdrawal threshold. At 6 h (*p* < 0.001 at 100 mg/kg (84.97 ± 2.14 g) and 200 mg/kg (86.21 ± 1.09 g)) and 24 h (*p* = 0.006 at 100 mg/kg (52.64 ± 1.67 g), *p* < 0.001 at 200 mg/kg (60.23 ± 0.85 g)), the response to mechanical stimuli increased compared to the pain model. After 48 h, the pain rebounded to the pre-injection level in all dosage groups.

### 2.3. The Effects of Tranilast in Regard to Mechanical Stimuli Post IONC Lasted up to 24 h in Comparison to Carbamazepine

**Figure 3:**
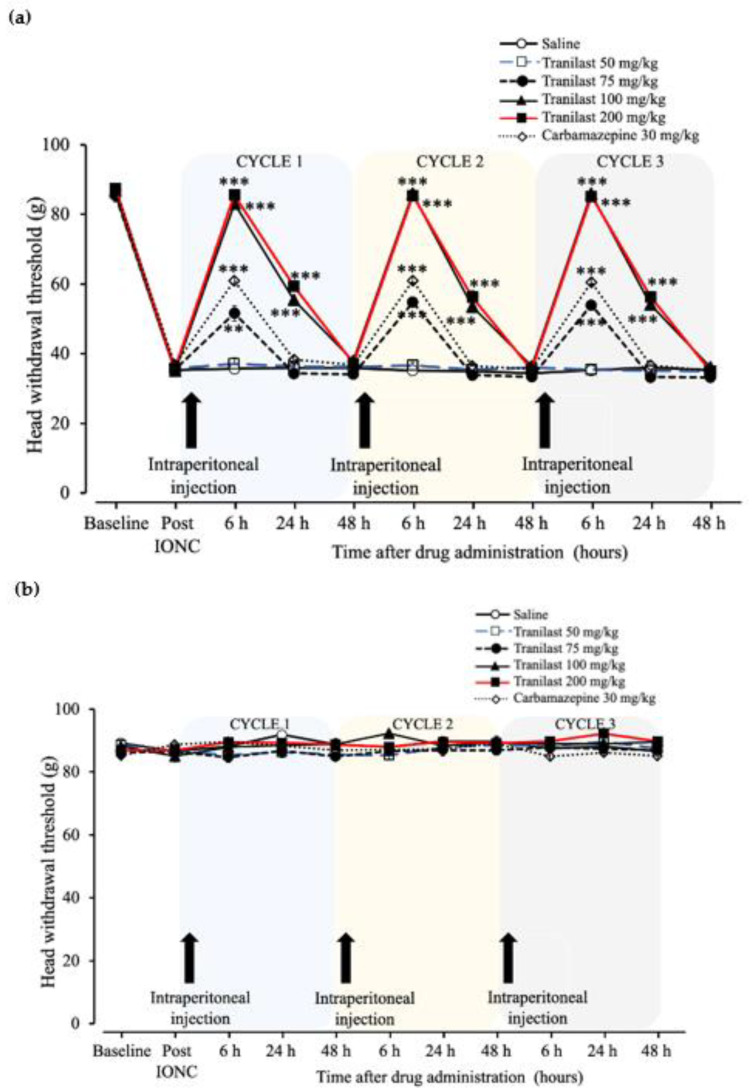
Head withdrawal threshold to mechanical stimuli after intraperitoneal injection of tranilast, carbamazepine or saline. (**a**) Ipsilateral side head withdrawal threshold in the neuropathic pain model following intraperitoneal administration of tranilast (50–200 mg/kg), carbamazepine (30 mg/kg) or saline injection. The experiment outcome is shown in three repetitive cycles. The behavioral responses to mechanical stimuli of the tranilast and carbamazepine groups are compared to the saline group. Doses of 100 and 200 mg/kg of tranilast showed increased head withdrawal thresholds at 6–24 h. Tranilast 75 mg/kg and carbamazepine (30 mg/kg) showed increased thresholds at 6 h and thereafter showed a rebound in threshold similar to that in the saline group. Tranilast 50 mg/kg did not show any changes in response to mechanical stimuli. (**b**) The contralateral side head withdrawal threshold showed no changes. The head withdrawal thresholds are represented as mean ± SEM. *n* = 6 per group. ** *p* < 0.01, *** *p* < 0.001, repeated measures analysis of variance followed by Tukey’s HSD test.

The head withdrawal threshold after intraperitoneal injection of tranilast (50, 75, 100 and 200 mg/kg), carbamazepine (30 mg/kg) or saline was recorded for three consecutive cycles (Figure 3a,b). An increased head withdrawal threshold on the ipsilateral side of the IONC was observed in the tranilast and carbamazepine groups at 6 h post-injection (Figure 3a, (F _(6,35)_ = 214.67), *p* < 0.001). The tranilast 75 mg/kg (51.62 ± 2.19 g) (*p* = 0.002), tranilast 100 mg/kg (83.13 ± 0.88 g) (*p* < 0.001), tranilast 200 mg/kg (85.56 ± 1.09 g) (*p* < 0.001) and carbamazepine groups (60.93 ± 0.72 g) (*p* < 0.001) showed increased tolerance to mechanical stimuli, while tranilast 50 mg/kg (37.07 ± 1.72 g) (*p* = 0.995) did not show any effect when compared to the saline group. At 24 h, the effect of the drug continued to last and showed an increased head withdrawal threshold on the ipsilateral side of the IONC in the tranilast group only (Figure 3a, F _(6,35)_ = 249.62), *p* < 0.001). Tranilast 100 mg/kg (55.48 ± 0.60 g) (*p* < 0.001) and 200 mg/kg (59.34 ± 0.85 g) (*p* < 0.001) showed increased tolerance to mechanical stimuli, while carbamazepine (*p* = 0.821), 75 mg/kg (34.34 ± 0.99 g) (*p* = 0.970) and 50 mg/kg (36.33 ± 0.92 g) (*p* = 1.00) did not show any effect when compared to the saline group. At 48 h, there was a drop in the head withdrawal threshold to the pre-injection level in all groups, which was similar in the saline group (Figure 3a). A similar pattern of results was observed for cycles 2 and 3. The contralateral side did not show any significant changes in any group (Figure 3b).

### 2.4. Effective Dose Required to Produce a 50% Response

**Figure 4:**
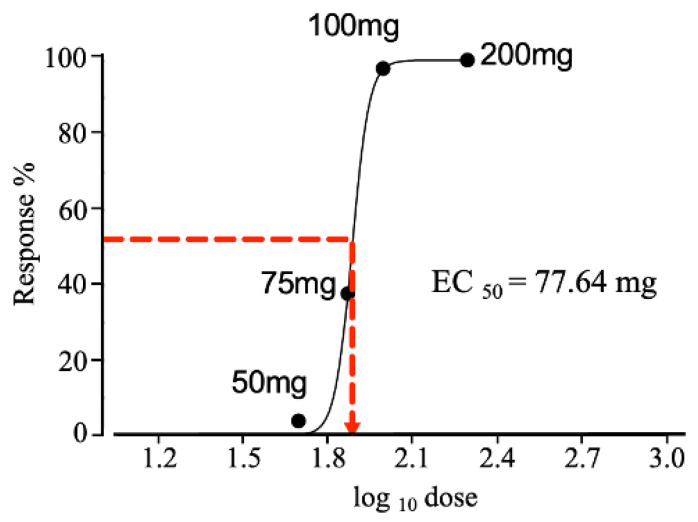
The s-shaped curve showing the dose-response relationship. The dose of tranilast is plotted as the log10 dose on the *x*-axis. The effect of the drugs as responses are plotted on the *y*-axis. The effect of tranilast (pain threshold) increased with increasing doses. The effects of tranilast increased up to 100 mg, and no further increases were seen beyond 100 mg, as 200 mg also showed the same response. The effective dose with which to achieve 50% of the maximum response (EC_50_) was determined to be 77.64 mg.

The responses to different dosages of tranilast (50–200 mg/kg) are plotted in Figure 4. The dose-response relationship is represented as an ‘s-shaped’ curve. The maximum effect of the drug was observed at 100 mg, and there was no further increase in the response, with a similar effect observed at 200 mg. The drug concentration at which 50% or half the effect was elicited is represented as EC_50_. The EC_50_ for tranilast was seen at a dosage of 77.64 mg.

### 2.5. Rotarod Performance Was Unaffected in the Tranilast Group and Motor Coordination Deficits Were Confirmed in the Carbamazepine Group in IONC Rats

**Figure 5:**
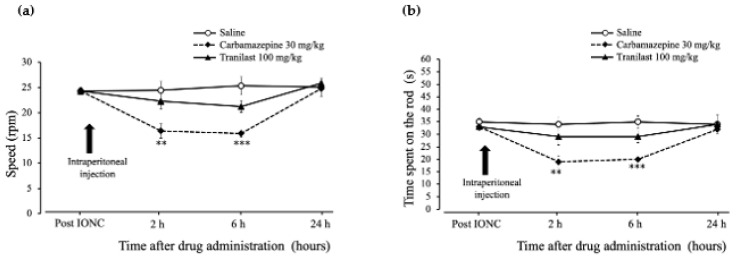
Rotarod performance test. (**a**) Changes in speed (rotations per minute (rpm)) and (**b**) change in time spent on the rod (seconds (s)) following intraperitoneal tranilast (100 mg/kg), carbamazepine (30 mg/kg), or saline injections at different times (2, 6 and 24 h) in the neuropathic pain model. Reduced speed and time spent on the rod were observed in the carbamazepine group compared to the saline group at 2 and 6 h. No significant changes are seen in the tranilast group. Values are presented as mean ± SEM. *n* = 6 per group. ** *p* < 0.01, *** *p* < 0.001, repeated measures analysis of variance followed by Tukey’s HSD test.

The baseline rotarod performance test was recorded seven days after IONC (24.28 ± 0.7 rpm). The speed (rotations per minute (rpm)) at which the rats remained on the rod showed significant changes 2 h (Figure 5a, (F _(2,15)_ = 6.83), *p* = 0.008) after drug injection. The performance of the carbamazepine group (16.39 ± 1.41 rpm) dropped significantly (*p* = 0.007), while there was no change in the tranilast group (22.28 ± 1.58 rpm) (*p* = 0.612) when compared to the saline group (24.45 ± 1.79 rpm). At 6 h, similar results were observed (Figure 5a, (F _(2,15)_ = 13.10), *p* = 0.001), and the performance of the carbamazepine group (15.89 ± 0.75 rpm) (*p* < 0.001) continued to be reduced, while no change was observed in the tranilast group (21.22 ± 1.16 rpm) (*p* = 0.100) when compared to the saline group (25.33 ± 1.8 rpm). At 24 h, there were no significant differences between the groups (Figure 5a, (F _(2,15)_ = 0.26), *p* = 0.778). With regard to the time (in seconds (s)) spent on the rod before the fall, there were significant changes at 2 h (Figure 5b, (F _(2,15)_ = 7.125), *p* = 0.007), and the carbamazepine group (19 ± 2.4 s) had significant time decreases (*p* = 0.006), while no change was observed in the tranilast group (29 ± 3.2 s) (*p* = 0.443) when compared to the saline group (34 ± 2.7 s). At 6 h, similar results were observesd (Figure 5b, (F _(2,15)_ = 19.499), *p* < 0.001), and the performance of the carbamazepine group (20 ± 0.7 s) (*p* < 0.001) continued to be reduced, while no change was observed in the tranilast group (29 ± 2.3 s) (*p* = 0.067) when compared to the saline group (35 ± 2.4 s). At 24 h, there were no significant differences between the groups (Figure 5b, (F _(2,15)_ = 0.404), *p* = 0.675). These observations are indicative of motor coordination deficits in the carbamazepine group and no apparent impact in the tranilast group.

### 2.6. BH4-Related Gene Expression Is Markedly Upregulated in Trigeminal Ganglions after IONC

**Figure 6:**
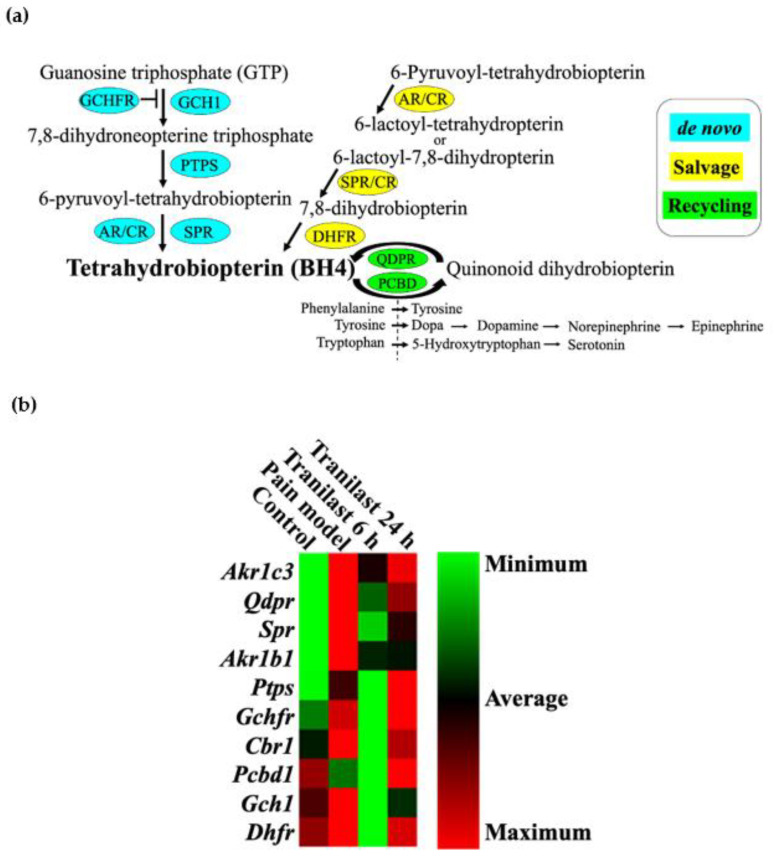
Tetrahydrobiopterin (BH4) synthesis pathway and analysis. (**a**) The three metabolic pathways involved in BH4 production, the de novo (blue), salvage (yellow), and recycling pathway (green). The synthesis of BH4 begins at the “de novo” pathway, starting from guanosine triphosphate (GTP) catalyzed by the following enzymes: GTP cyclohydrolase 1 (GCH1) modulated by the GTP cyclohydrolase 1 feedback regulator (GCHFR), 6-pyruvoyltetrahydropterin synthesis (PTPS), and sepiapterin reductase (SPR). Additionally, the final step can be influenced by two enzymes: aldoketo reductase (AR) and carbonyl reductase (CR). Alternatively, the “salvage pathway” involves SPR and dihydrofolate reductase (DHFR). The “recycling pathway” involves the regeneration of BH4 from quinonoid dihydrobiopterin (BH2) with the following enzymes: pterin-4α-carbinolamine dehydratase (PCBD) and quinoid dihydropteridine reductase (QDPR). (**b**) Cluster heatmap of BH4 synthesis visualized under different conditions: pain model (infraorbital nerve constriction (IONC) + no treatment) and tranilast-treated group at the 6th and 24th hours (IONC + tranilast treatment) compared to the control group. The heat map shows the normalized 2^−∆^^∆CT^ values. *n* = 6 per group.

We custom-made an RT^2^ profiler PCR array covering all genes involved in the synthesis of BH4. Ten genes involved in the de novo, salvage and recycling pathways in BH4 biosynthesis were analyzed using the collected TG tissues (Figure 6a). The array included guanosine triphosphate cyclohydrolase 1 (*Gch1*), GTP cyclohydrolase 1 feedback regulator (*Gchfr*), 6-pyruvoyltetrahydropterin synthesis (*Ptps*), sepiapterin reductase (*Spr*), aldoketo reductase (*Akr*-*Akr1c3* and *Akr1b1*), carbonyl reductase (*Cbr*), dihydrofolate reductase (*Dhfr*), pterin-4α-carbinolamine dehydratase (*Pcbd*) and quinoid dihydropteridine reductase (*Qdpr*). These selected genes were analyzed under four different conditions following nerve injury i.e., pain model (IONC + no treatment) 6 and 24 h post-tranilast injection and compared to the control group. Heat map analysis revealed an increase in the magnitude of gene expression in the pain model. The tranilast treated group at 6 h showed a clearer pattern of downregulation, and, in contrast, upregulation was observed at 24 h. Collectively, these findings confirmed the role of genes involved in BH4 production in neuropathic pain (Figure 6b).

### 2.7. The Expression of Spr and Akr Increases in Trigeminal Ganglions of Nerve Injury Rat Models and Decreases after Tranilast Treatment

**Figure 7:**
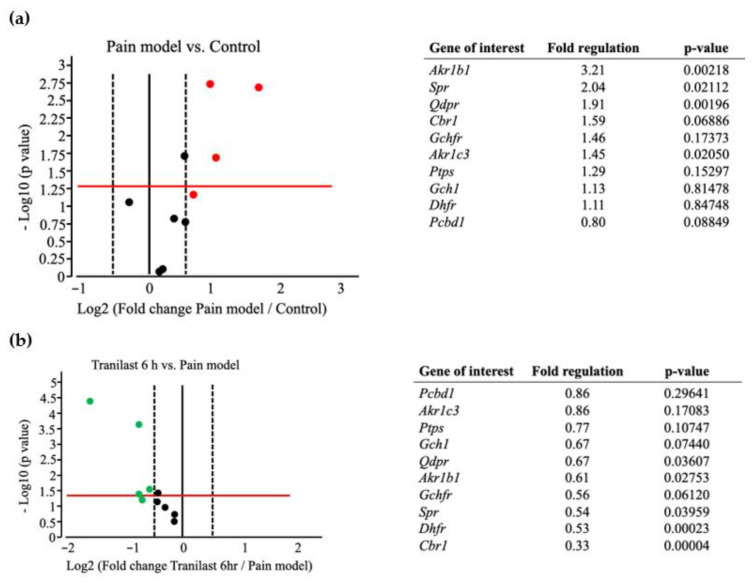

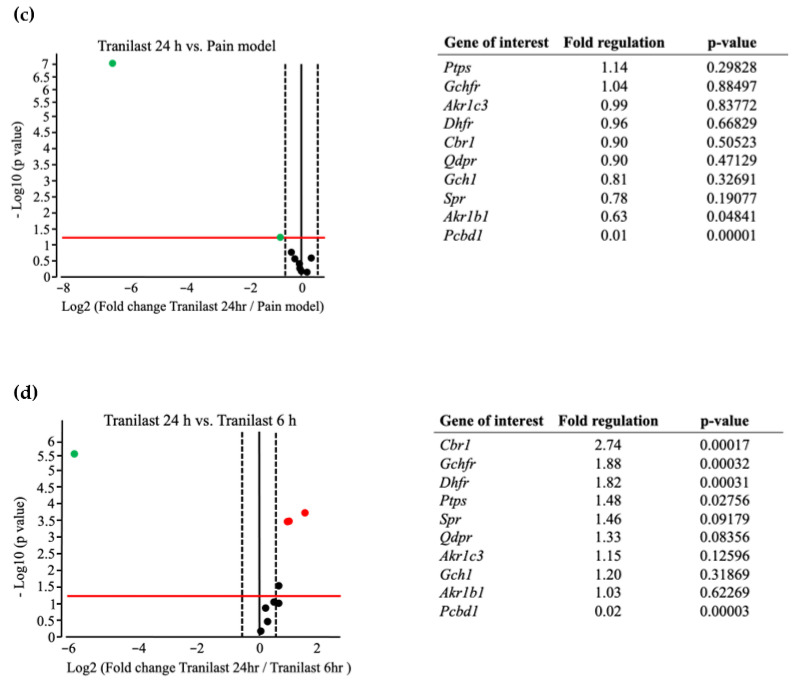
Fold regulation of genes involved in the BH4 pathway from the RT^2^ profiler PCR array data, represented as a volcano plot. The red horizontal line represents *p* = 0.05, the points above are indicative of *p* < 0.05, and the points below are indicative of *p* > 0.05. The black vertical lines represent the log2 fold change threshold of −0.5/0.5 respectively, with no change indicated by the middle line. Red, black, and green dots indicate upregulated, no change and downregulated genes, respectively. The dots above the red line indicate a greater than 1.5-fold change (*x*-axis) and *p* < 0.05 (*y*-axis). The fold regulation and *p*-values are listed beside each volcano plot. (**a**) Volcano plot between the pain model (infraorbital nerve constriction (IONC) + no treatment) and the control group. (**b**) Volcano plot between the tranilast-treated group after 6 h (IONC + tranilast treatment) and the pain model (IONC + no treatment). (**c**) Volcano plot between the tranilast-treated group after 24 h (IONC + tranilast treatment) and the pain model (IONC + no treatment). (**d**) Volcano plot between the tranilast-treated group after 24 h and 6 h (IONC+ tranilast treatment). Fold regulation cut off: 1.5; *p*-value cut off = 0.05; *n* = 6 per group.

In the pain model (IONC + no treatment), the expression of four genes involved in BH4 synthesis was upregulated (more than 1.5-fold change) compared to the control group (no IONC, no treatment) (Figure 7a). Significantly upregulated (> 1.5-fold change, *p* < 0.05) genes included *Akr1b1* (fold regulation = 3.21, *p* = 0.0021) and *Spr* (fold regulation = 2.04, *p* = 0.0211). This increased expression demonstrates further understanding of the contribution of *Spr* and *Akr* in the production of BH4 in neuropathic pain.

After tranilast injection, 6 h later (IONC+ tranilast treated at 6 h), a significant downregulation of four genes (<1-fold change, *p* < 0.05) was noted when compared to the pain model (Figure 7b). Of these, *Akr1b1* (fold regulation = 0.61, *p* = 0.0275) and *Spr* (fold regulation = 0.54, *p* = 0.0395) were notable. They were highly expressed in the pain model and were subsequently downregulated following tranilast treatment.

Furthermore, the expression of *Akr1b1* (fold regulation = 0.63, *p* = 0.0484) remained significantly downregulated (<1-fold change, *p* < 0.05) even 24 h after tranilast injection (Figure 7c). Additionally, *Pcbd1* was also downregulated (fold regulation = 0.01, *p* = 0.00001) compared to the pain model. On comparison of 24 h to 6 h, upregulation of *Cbr1* (fold regulation = 2.74, *p* = 0.0001) and *Gchfr* (fold regulation = 1.88, *p* = 0.0003) was observed. The expression of *Spr* and *Akr1b1* was also slightly increased, though there was no significant change, which could explain the rebound increase in BH4 production (Figure 7d).

## 3. Discussion

Orofacial neuropathic pain has been studied more extensively in recent decades to better understand the complexity of the condition and develop an effective treatment [3]. Several animal models have been developed to mimic the orofacial neuropathic pain state in humans, to help understand the complex mechanism and to develop appropriate therapeutic interventions [7]. The prototype model for trigeminal neuralgia is a chronic constriction injury of the infraorbital nerve, which has been the most extensively utilized since 1994 [18]. In this type of constriction model, the maxillary branch of the trigeminal nerve is ligated, thereby causing pain-associated behaviors in the whisker pad area, such as hypersensitivity to mechanical stimuli [19]. The effectiveness of this IONC model in mimicking chronic neuropathic pain has been demonstrated in our previous studies as well [20,21]. In the present study, we used a similar neuropathic pain model to validate the analgesic effect of tranilast.

The neuropathic pain model was created by constriction of the trigeminal nerve via infraorbital nerve ligation in rats. The mechanical sensitivity of the rats (whisker pad area) following IONC was assessed seven days after nerve injury to mimic chronic pain [22]. The pain threshold in response to mechanical stimuli was significantly reduced on the ipsilateral side of the injury. Graded intraperitoneal injections of tranilast were administered (50–200 mg/kg), and the dosage was calculated from previous reports [23,24]. Our findings revealed a dose-dependent reduction in pain. At 6 h post-drug administration, significant changes in the head withdrawal threshold were observed at doses ranging from 75 to 200 mg/kg, with 100 and 200 mg/kg remaining effective for up to 24 h. Additionally, we observed the higher dose of 200 mg/kg compared to 100 mg/kg does not cause any further increase in its effects, indicating it has reached a plateau/ceiling effect. These findings demonstrate the analgesic effects of tranilast. Similar results of decreased response to mechanical stimuli were seen in an earlier report after tranilast administration to the whisker pad skin [25].

Considering the above results, we aimed to replicate the experimental conditions numerous times to ensure that the results were valid. We decided to perform three cycles of similar drug interventions (i.e., intraperitoneal injections of 50–200 mg/kg), wherein the intraperitoneal injections were repeated 48 h after the initial dose. Interestingly, we observed a consistent trend in outcomes throughout all cycles. Furthermore, we compared the effects of tranilast with the “gold standard” drug, carbamazepine, which is the first line of choice in the current treatment of neuropathic pain [26]. The dosage of carbamazepine was 30 mg/kg based on previous experimental models [27]. The head withdrawal thresholds of the tranilast (100 and 200 mg/kg) groups were greater than that of carbamazepine 6 h after drug administration. In addition, the carbamazepine group exhibited a rebound in pain, but the tranilast (100 and 200 mg/kg) groups were pain-free for 24 h.

The current literature on the therapeutic potential of tranilast for pain relief has revealed only one study in which pain caused by endometriosis was relieved [13]. Our findings from the behavior testing emphasize the therapeutic potential of tranilast in neuropathic pain. The effective concentration of tranilast from our observations was calculated to determine the “half-maximum effective concentration” (EC_50_). The dose-response curve was plotted, and the EC_50_ was estimated as 77.64 mg. This represents the concentration of tranilast required to cause half of the maximum possible effect.

Tranilast is a clinically approved drug and has been in use since the 1980s in Japan, South Korea and China [28]. Several reports have suggested that tranilast has no side-effects and is well-tolerated by patients [10,12]. It has been in clinical use for over 40 years and has been effectively used in the treatment of asthma, hypertrophic scars, and allergies without causing adverse side-effects [29]. Carbamazepine, despite being the primary choice for treating neuropathic pain, adverse effects, particularly those related to long-term usage, require the development of a comparable alternative. The commonly reported side-effects include dizziness, drowsiness and nausea [30]. Dizziness is especially troublesome and affects the daily activities of patients.

Motor coordination in rodents has been widely tested using rotarod performance tests. The rotarod test measures motor activity and aids in evaluating drug effects [31]. In this study, we used the accelerating rotarod to assess motor coordination and balance in rats after the administration of tranilast and carbamazepine. The rats were trained for a few days to remain on the rotating and accelerating rotarods before the final observation. To exclusively evaluate the effects of the drugs, we recorded the baseline values seven days after the nerve injury to rule out any nerve-injury-induced changes (pain-related behaviors) [32]. The carbamazepine group showed a significant drop in the retention time on the rod 2 and 6 h after drug administration. Similar results were observed in a previous study as well [33]. These results signify impairment in motor coordination induced by carbamazepine. In contrast, the tranilast group showed only minor numerical changes, and there was no significant drop in the retention time on the rod compared with that of the saline-injected group. Our findings suggest that tranilast does not impair motor coordination.

Furthermore, to better understand the exact mechanism underlying the effectiveness of this drug in treating neuropathic pain, we performed molecular analysis of the target tissue, the TG tissues. The TG is the sensory ganglion of the trigeminal nerve and modulates pain transmission in the orofacial region. Several previous studies have investigated the TG to better understand orofacial nociception [20,21,34].

In this study, we analyzed 10 genes related to BH4 synthesis. Those involved in the three pathways, namely the de novo, salvage and recycling pathways, were analyzed in the collected TG tissues. A series of studies have also indicated a link between chronic pain and BH4 upregulation [35,36,37,38,39]. A notable feature of the BH4 pathway is that even though SPR is targeted to reduce the levels of BH4, the residual levels are well-maintained by the recycling pathway, and no adverse effects related to cardiovascular or neurological effects have been reported [40,41].

Cluster heat map analysis of the genes involved in the BH4 pathway showed an interesting pattern in the magnitude of gene expression. We observed an upregulation of the genes (particularly *Gch1*, *Gchfr*, *Spr*, *Akr*, *Dhfr* and *Cbr*) in the pain model (IONC + no treatment) compared to the control. In contrast, downregulation of the same genes was observed 6 h post-injection of tranilast, and further upregulation of these genes 24 h post-injection was also seen. This pattern of expression of BH4 related genes coincides with our principal finding of pain alleviation in the behavioral analysis test at 6 h after tranilast injection and a rebound of pain after 24 h. These findings are also in accordance with previous reports of elevated BH4 levels in peripheral nerve injury [35].

Increased expression of *Spr* and *Akr* was evident in the neuropathic model, which was effectively downregulated after intraperitoneal injection of tranilast (6 h). *Spr* encodes the enzyme SPR, which is one of the key enzymes in the biosynthesis of BH4, involved in both the de novo and the salvage pathway. Additionally, AR, CR and DHFR are involved in BH4 metabolism. Although several enzymes and cofactors are involved in the synthesis of BH4, SPR plays a pivotal role, as it is the terminal enzyme. Previously, researchers have tried to explore SPR inhibitors; however, none have currently been established for use in treating neuropathic pain [42]. SPRi3, a synthetic derivative, is under trial for the reduction of neuropathic and inflammatory pain. However, it has not yet been approved for clinical use [35].

We hypothesized that the systemic administration of tranilast would reduce neuropathic pain. This was also evident in our observations. Behavioral tests indicated the analgesic effect of tranilast. In the PCR array analysis, there was increased expression of genes regulating BH4 synthesis in the pain model, which decreased after the administration of tranilast. Tranilast has been approved for clinical use for more than four decades and is effectively used as an anti-allergic drug. Although it has been in clinical use for several decades, the effectiveness of this novel drug in treating neuropathic pain has not been reported. From the results of the current study, we demonstrated a new dimension in the therapeutic potential of tranilast. To the best of our knowledge, this is the first study to validate the effectiveness of tranilast in treating neuropathic pain in an animal model and analyze the changes in BH4 synthesis. Hence, it can be effectively implemented in clinical practice to treat neuropathic pain.

## 4. Materials and Methods

### 4.1. Animals

**Figure 8:**
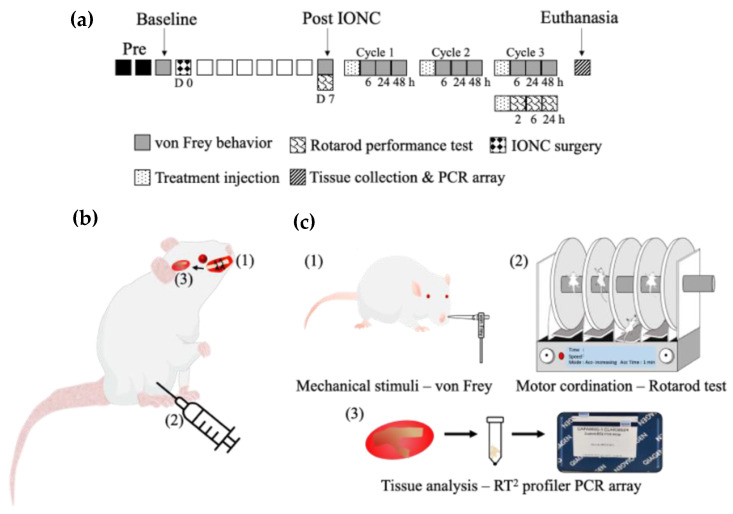
Scheme for the experiment timeline, neuropathic pain model, and methods. (**a**) The experiment timeline showing the procedures in the neuropathic pain model. (D: Day, IONC: Infraorbital nerve constriction). (**b**) Neuropathic pain model, (1) IONC, (2) treatment injection—tranilast/carbamazepine/saline, (3) trigeminal ganglion tissue. (**c**) Evaluation tests conducted: (1) pain behavior tested as a response to mechanical stimuli using the von Frey test; (2) motor coordination assessed with the rotarod performance test; (3) trigeminal ganglion tissues analyzed using an RT^2^ profiler PCR array.

Male Sprague Dawley rats (4–9 weeks old) were used for all the experiments (CLEA Japan, Osaka, Japan). The rats were housed in groups of two per cage under a controlled 12 h light/dark cycle, at 18 to 23 °C with 40 to 60% humidity condition, received regular food chow and water available ad libitum. All efforts were made to reduce suffering and to minimize the number of animals used. All experimental procedures were conducted in accordance with the guidelines of the Animal Research Committee of Tokushima University, Japan (Protocol number: T30-75 and T2020-108) and the International Association for the Study of Pain. All tests were conducted in a randomized, blinded, controlled manner. The experimental timeline, neuropathic pain model, and methods are schematically shown in Figure 8. The sample size was calculated based on the preliminary sample data using G power 3.1 version. A sample size of six per group was estimated to provide 95% power, α error of 0.05 and effect size of 1.05.

### 4.2. IONC

The IONC surgery was performed on one side as described previously [19,20]. Rats were deeply anesthetized before the surgical procedure with 0.375 mg/kg medetomidine (Nippon Zenyaku Kogyo Co., Ltd., Fukushima, Japan), 2.5 mg/kg butorphanol (Meiji Seika Pharma Co., Ltd., Tokyo, Japan) and 2 mg/kg midazolam (Sandoz K.K., Yamagata, Japan). All incisions were made intraorally such that they did not affect behavior testing in the whisker pad area. An approximately 1-cm-long incision was made proximal to the first molar, along the gingivobuccal margin. The infraorbital nerve, which is the terminal branch of the maxillary division of the trigeminal nerve, was exposed. For about 0.5 cm, the surrounding attached tissues were freed. Two ligatures (4-0 silk sutures) were tied loosely around the nerve to create constriction injury. The contralateral infraorbital nerve was intact.

### 4.3. Intraperitoneal Injection of Drugs to the IONC Model

Seven days after the IONC surgery, intraperitoneal injection of tranilast (Kissei Pharmaceutical, Nagano, Japan) (50–200 mg/kg in PBS), carbamazepine (FUJIFILM Wako Pure Chemical Corporation, Osaka, Japan) (30 mg/kg in DMSO), or saline was injected. The total volume of the injected drug was 1 mL.

### 4.4. Behavior Test to Assess Mechanical Sensitivity

Isoflurane (2.5% inhalation) was used to mildly anesthetize the rats, and their whiskers were shaved using clippers one day prior to the behavior assessments [43]. The rats were restrained in Durham Animal Holders (37100, Ugo Basile, Varese, Italy), which are routinely used for orofacial stimulation in rats. The holder had a semicircular hole (diameter: 7 cm, height: 2 cm) that allowed the snout to protrude and unrestricted withdrawal of the snout during application of the mechanical stimulus. An electronic von Frey anesthesiometer (Model 1601C, IITC Instruments, Woodland Hills, CA, USA) was used to apply stimuli to the center of the whisker pad area. The force (g) applied when the head was withdrawn was recorded. Mechanical stimuli were applied to both the ipsilateral and contralateral sides. The behavioral assessment was performed five times on either side alternatively, with an interval of 1 min between stimulations. For each side, five datasets were recorded, and the results were averaged after the highest and lowest values were excluded.

Behavioral assessment was performed one day prior (baseline) to the IONC surgery, seven days after IONC surgery, and repeated every 6, 24 and 48 h after the intraperitoneal injections of the drugs. The rats were administered with carbamazepine (30 mg/kg), tranilast (50, 75, 100 or 200 mg/kg) or saline. Behavioral assessment was performed by an examiner who was blinded to the experimental groups.

### 4.5. Rotarod Performance Test to Assess Motor Coordination

A rotarod performance test was conducted to assess the motor coordination of the rats after the drug administration. This test evaluates both the time (in s) the rat spends on the rotating rod and the speed (rpm) at which it maintains its balance before falling. The instrument (LE 8500, Harvard Apparatus, MA, USA) comprised a rotating rod with a diameter of 60 mm, and the rats were placed on the rod facing the direction of rotation. This methodology was adapted from previous reports [31]. Prior to the actual test, the rats were trained for two days. The rotating rod was set to gradually accelerate from 4 to 40 (rpm) over one minute. The latency (time in s) and speed (rpm) before the fall were recorded (i.e., the test stopped automatically when the rat fell off the rod). Additionally, if the rat turned around (passive running) or restrained itself by holding on to the rod by just rotating, the test was stopped. The pre-drug time spent on the rod, that is, the baseline time, was recorded after the IONC surgery. The post-drug time spent on the rod was recorded at 2, 6 and 24 h after the intraperitoneal drug administration. Rats were injected with carbamazepine (30 mg/kg), tranilast (100 mg/kg) or saline (control). Five sets were recorded for each trial, and the results were averaged after eliminating the highest and lowest values.

### 4.6. BH4 Pathway RT^2^ Profiler PCR Array

TG tissues were collected from the control and treatment groups (pain model (i.e., IONC with no treatment) and tranilast 100 mg/kg at 6 and 24 h). Each experiment was repeated six times. Total RNA was isolated from the homogenized tissue using an RNeasy Plus Mini Kit (Cat. No. 74134, Qiagen, Venlo, The Netherlands) according to the manufacturer’s guidelines. For cDNA conversion, 1 µg of isolated RNA was used for all samples using the RT^2^ First Strand Kit (Cat. No. 330404, Qiagen, Venlo, The Netherlands). Genomic DNA elimination was also performed for all samples. The resultant cDNA was further used to perform real-time PCR using a Custom RT^2^ PCR array (96 wells) for the rat BH4 signaling pathway (Cat. No CAPA9600-1: CLAR38524, Qiagen, Venlo, The Netherlands) with RT^2^ SYBR Green (Cat. No. 330524, Qiagen, Venlo, The Netherlands). The cycling conditions were as follows: 1 cycle of 10 min at 95 °C, followed by 45 cycles of 15 s at 95 °C, and 1 min at 60 °C. The C_T_ cutoff was set at 35. The fold-change was then calculated using the 2^−∆∆CT^ formula. The fold regulation cut off (1.5) and *p*-value cut off (0.05) were set by referring to the previous research [44]. For the analysis, we customized 10 genes, and the genes involved in the synthesis of BH4 were all included in the array.

### 4.7. Statistical Analysis

The von Frey behavior and rotarod data are presented as the mean ± standard error of the mean (SEM). Data were analyzed using repeated-measures analysis of variance followed by Tukey’s HSD test. Statistical analyses were performed using SPSS for Windows (ver. 27, IBM, Tokyo, Japan). The dosage curve (EC_50_) was plotted using GraphPad Prism 9 (GraphPad Software, La Jolla, CA, USA). Qiagen’s Gene Globe Data Analysis Center was used to interpret the PCR data (RT^2^ profiler array). The *p*-values for each gene in the treatment and control groups were computed using the Student’s *t*-test on the replicate 2^−∆∆CT^ values.

## Data Availability

The datasets generated and analyzed during the current study are available from the corresponding author on reasonable request.

## Institutional Board Review

The study was conducted according to the Declaration of Helsinki. All protocols were approved by the Ethics Committee of Tokushima University (protocol numbers: T30-75, approval date 27 September 2018; and T2020-108, approval date 1 May 2021).
